# Upper Urinary Tract Urothelial Carcinoma With Squamous, Glandular, and Sarcomatoid Variants in a Horseshoe Kidney: A Novel Case Report and Literature Review

**DOI:** 10.7759/cureus.19627

**Published:** 2021-11-16

**Authors:** Jehad Fikri, Abdullah M Almalki, Sattam A Almalki, Muradi Murad, Sara Makhdoum, Fadil Hassan

**Affiliations:** 1 Medicine, King Saud Bin Abdulaziz University for Health Sciences, Jeddah, SAU; 2 Pathology, King Abdulaziz Medical City, Jeddah, SAU; 3 Urology, King Abdulaziz Medical City, Jeddah, SAU

**Keywords:** transitional cell carcinoma, sarcomatoid renal cell carcinoma, fused kidney, urothelial malignancy, horse shoe kidney

## Abstract

Horseshoe kidney is a congenital anomaly, which consists of fusion of the lower poles of the kidneys. Cancer in a horseshoe kidney is common, possibly because of the increased risk of chronic obstruction, renal calculi, and recurrent urinary infection. We report a case of a 64-year-old male with a horseshoe kidney who presented to our hospital with gross hematuria and flank pain, which was highly suggestive of pyelonephritis. Comprehensive workup and imaging were performed and showed an extremely rare form of tumor consisting of three histological variants: squamous, glandular, and sarcomatoid. To the best of our knowledge, this is the first case reported with these three histological variants in a horseshoe kidney.

## Introduction

Upper urinary tract urothelial carcinomas (UCs) are considered rare tumors and account for only 5%-10% of all the UCs [1]. Most of the patients who are diagnosed with UC present with hematuria [2]. UC is discovered using computed tomography (CT) with contrast, and it most commonly appears as a filling defect in the collecting system [3]. Patients with upper urinary tract UC vary in terms of recurrence and survival rate, depending mainly on the grade and pathological stage of the tumor [4]. Horseshoe kidney is considered one of the most common congenital anomalies in the kidneys, with an incidence of approximately one in 600-800 individuals [5]. Renal pelvic UC occurs more frequently in cases with horseshoe kidneys than in the normal population [6]. In this report, we present a case of a horseshoe kidney diagnosed with upper urinary tract UC with three histological variants: squamous, sarcomatoid, and glandular. Furthermore, sarcomatoid variant is extremely rare to happen in horseshoe kidneys, and according to our search in PubMed, we have not come across any previously reported case. This report aims to present this rare case using theoretical concepts from our discipline and to share our approach in hope of achieving a better understanding of similar cases.

## Case presentation

**Figure 1:**
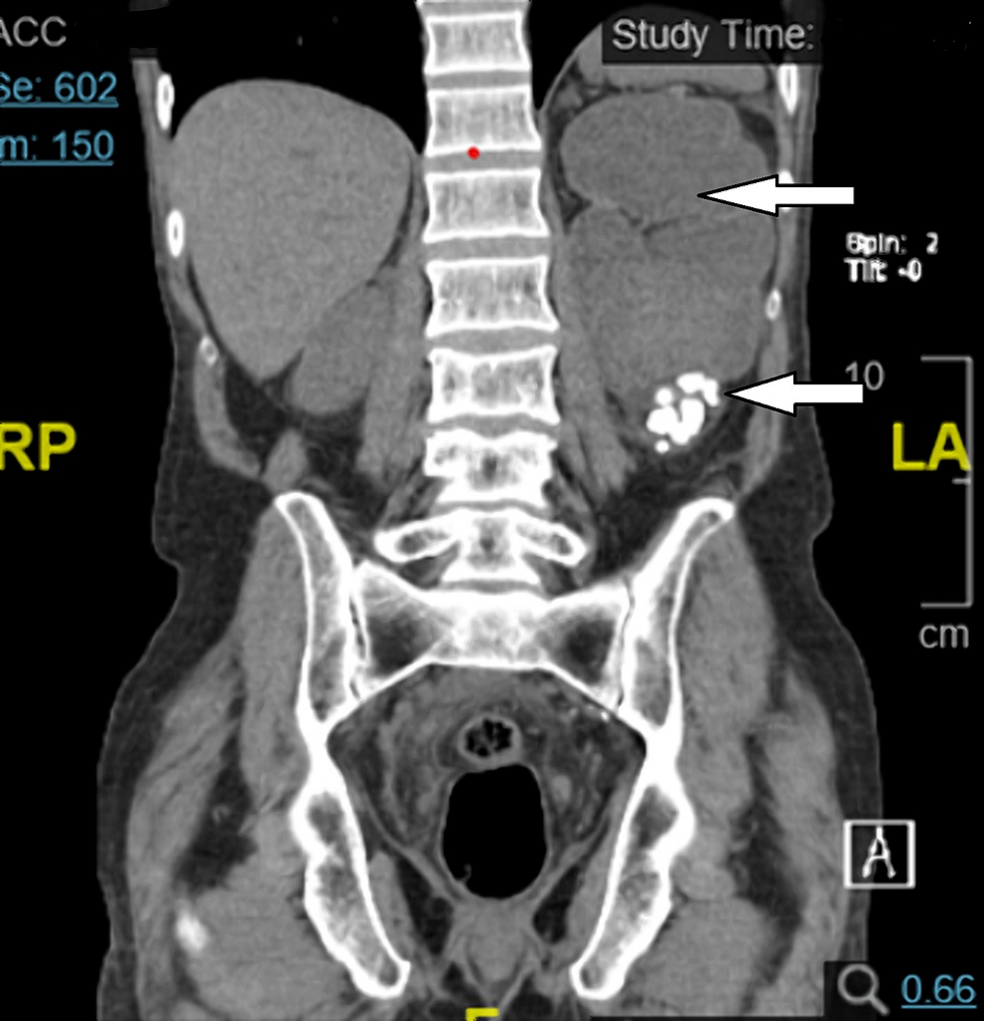
Coronal CT view of abdomen and pelvis displaying severe left hydronephrosis with multiple stones. CT: computed tomography.

**Figure 2:**
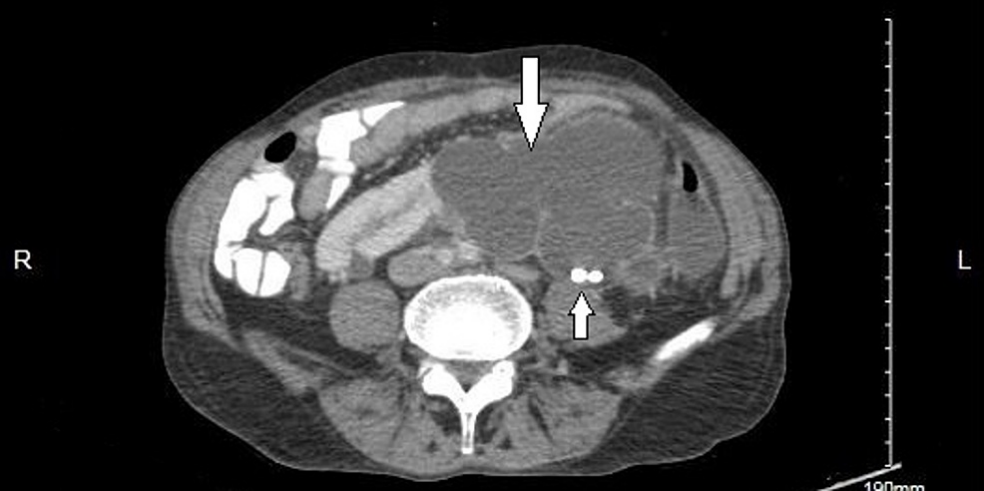
Axial CT view showing left kidney with severe hydronephrosis and multiple stones. CT: computed tomography.

**Figure 3:**
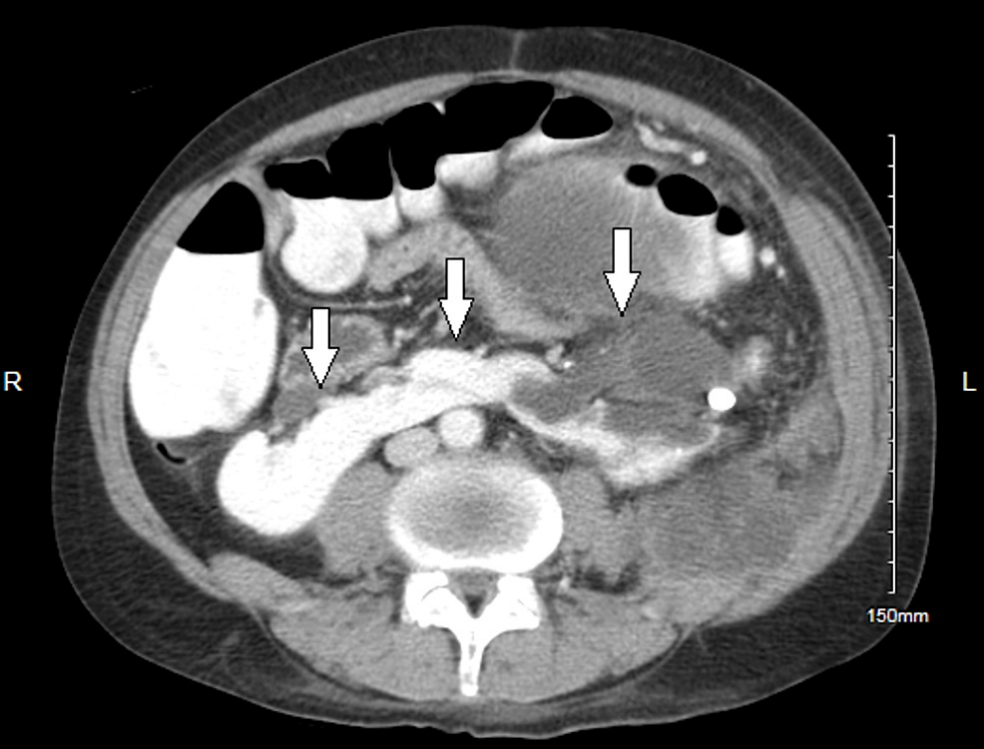
CT with contrast shows horseshoe kidney. CT: computed tomography.

A 64-year-old Caucasian male smoker with a horseshoe kidney with a history of open pyelolithotomy 18 years ago, presented to King Abdulaziz Medical City in mid-2020 with a report from another hospital stating that he developed gross hematuria six months prior, which was treated as a urinary tract infection. A CT of the abdomen and pelvis was performed in that hospital, showing a horseshoe kidney with severe left hydronephrosis and enlarged retroperitoneal lymph nodes, with the largest one located in the posterior part of the left renal artery measuring 4.7 × 3.5 × 2.6 cm. Additionally, there were multiple stones (Figures 1, 2, 3). Urine culture was performed and revealed that various organisms were isolated (10-100,000 CFU/ml). Urinalysis showed a small amount of blood with a moderate presence of leukocytes and a trace protein.

**Figure 4:**
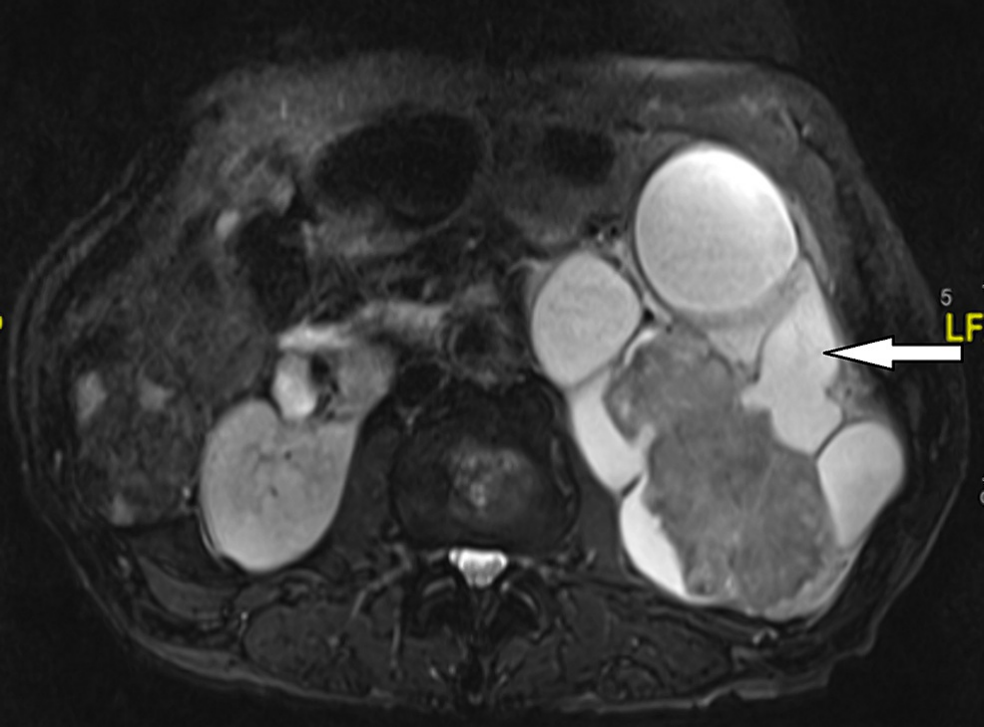
MRI shows hydronephrosis of the left kidney with a large mass within it centrally and further satellite lesions. MRI: magnetic resonance imaging.

At the end of 2020, the patient underwent magnetic resonance imaging (MRI). The MRI showed a horseshoe kidney with chronic hydronephrosis of the left kidney and a large mass within it centrally with further satellite lesions, which all likely represent UC and associated lymphadenopathy along the para-aortic chain (Figure 4). Additionally, a finding of chronic pancreatitis was noted with dilated duct and stone, for which the patient was referred to the gastroenterology department. Furthermore, a bone scan and chest CT were performed, and no significant abnormality or metastasis was found.

After a couple of days, the patient presented to the emergency department with non-radiating progressive lower abdominal and left colicky flank pain for three days with hematuria and constipation with fullness. The patient denied any history of fever or vomiting. There were no other genitourinary symptoms, scrotal pain, or change in the level of consciousness. Vital signs were measured and were as follows: blood pressure, 151/71 mmHg; heart rate, 109; respiratory rate, 20; and temperature, 37.1℃. The weight of the patient was 48.4 kg, and height was 166 cm. The chest was clear, while the abdomen was tender over the left side and the left flank area. Lab results were obtained (Table 1). Urine culture was performed and showed more than 100,000 CFU/ml of *Staphylococcus aureus* being isolated. Abdomen and pelvis CT was performed to rule out any intra-abdominal collections, but it was negative. The urology team was consulted for his chief complaint, as his case is already known to them. On examination, they found that the abdomen was soft with lower left quadrant tenderness. They concluded that the pain was less likely to be from the left kidney as the CT findings were stable. Furthermore, based on the urine culture result, the patient was prescribed Bactrim and was given an appointment for admission a week later to perform a flexible ureteroscopy with biopsy.

**Table 1:** Lab results. RBC: red blood cells; MCV: mean corpuscular volume; MCH: mean corpuscular hemoglobin; Hb: hemoglobin; HCT: hematocrit; WBC: white blood cells; BUN: blood urea nitrogen; ALT: alanine aminotransferase; AST: aspartate aminotransferase; ALP: alkaline phosphatase.

Labs	Values
RBC	4.3
MCV	79.2
MCH	26.3
HCT	34.4
Hb	11.4 g/dL
WBC	12.3
Neutrophils	10.77
Lymphocytes	0.65
Platelets	641
Preoperative creatinine	78 umol/L
Postoperative creatinine	61 umol/L
Sodium	135 mmol/L
Potassium	4.5 mmol/L
Chloride	104 mmol/L
BUN	3.5 mmol/L
ALT	15 U/L
AST	15 IU/L
ALP	252 U/L
Albumin	37 g/L
Total bilirubin	3.1 umol/L
Serum calcium	2.35 mmol/L

**Figure 5:**
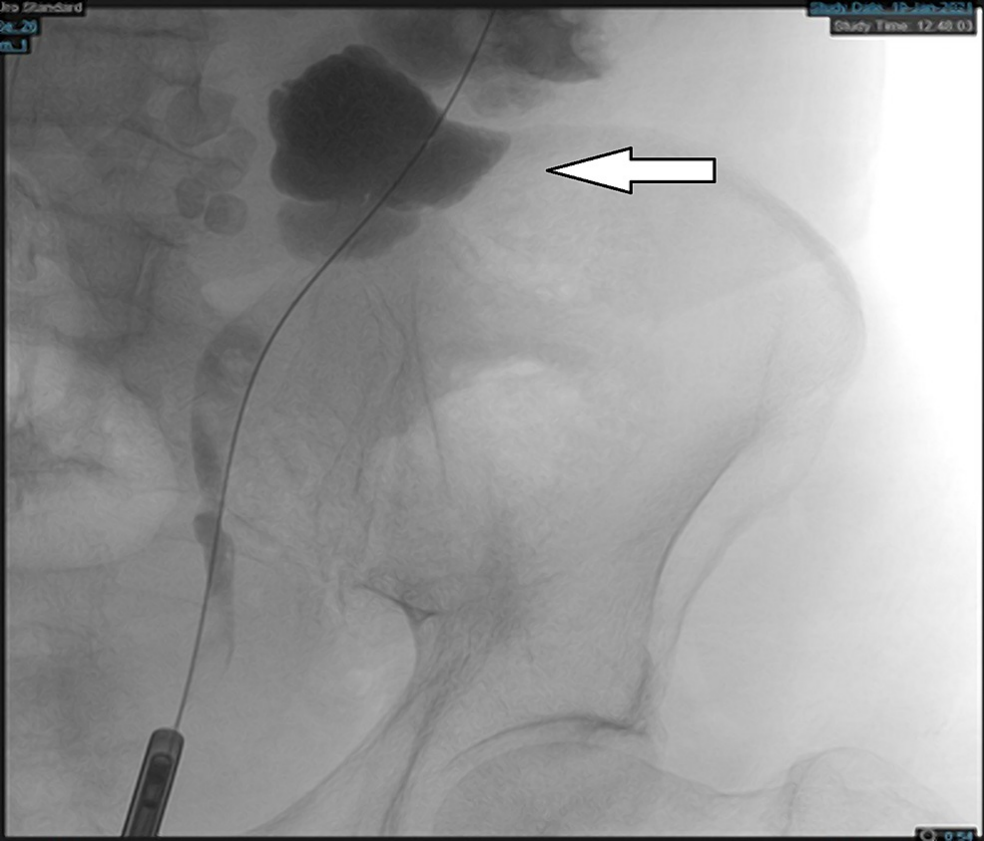
Retrograde pyelography shows that the left kidney is severely dilated.

After one week, the patient underwent ureteroscopy with cytology and culture based on his MRI findings. Cystoscopy and retrograde pyelography showed normal bilateral ureters. However, the left kidney was severely dilated, and cloudy urine was noticed from the left ureteric orifice. Thus, culture and cytology were taken; then, a double-J (DJ) stent was inserted (Figure 5). Urine culture was positive for *S. aureus*, and the patient was already on Bactrim. The urine cytology result was negative for high-grade UC, but benign urothelial cells, squamous cells, inflammatory cells, and red blood cells were present. Furthermore, since cancer could not be excluded, the patient was discharged and given an appointment for a left nephroureterectomy.

After two weeks, the patient presented to the emergency room complaining of left flank pain for five days with hematuria and constipation. The urology team was again consulted and suspected urinary tract infection at the site of the double J stent. Thus, the patient was admitted, given antibiotics, and was planned for the open left radical nephroureterectomy the following day. The surgery was successfully performed, and samples were sent to the histopathology department.

**Figure 6:**
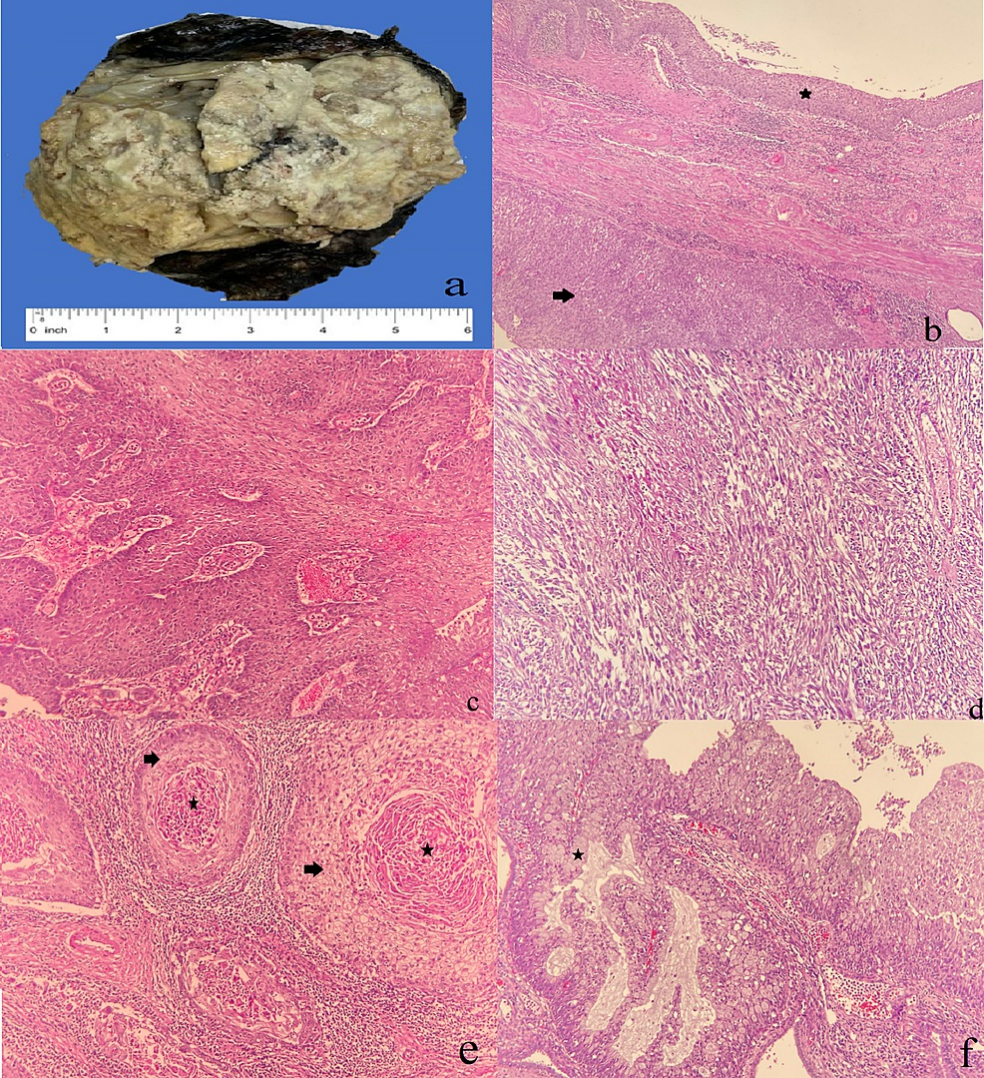
Gross and microscopic assessment of the mass. Kidney shows a fairly circumscribed bulging mass (a). Urothelial dysplasia (asterisk), with underneath solid growth of urothelial carcinoma (b). Urothelial carcinoma with papillary architecture (c). Sarcomatoid pattern (d). Squamous differentiation (arrowhead) and keratin pearls (asterisk) (e). Glandular differentiation and goblet cells lining (asterisk).

**Figure 7:**
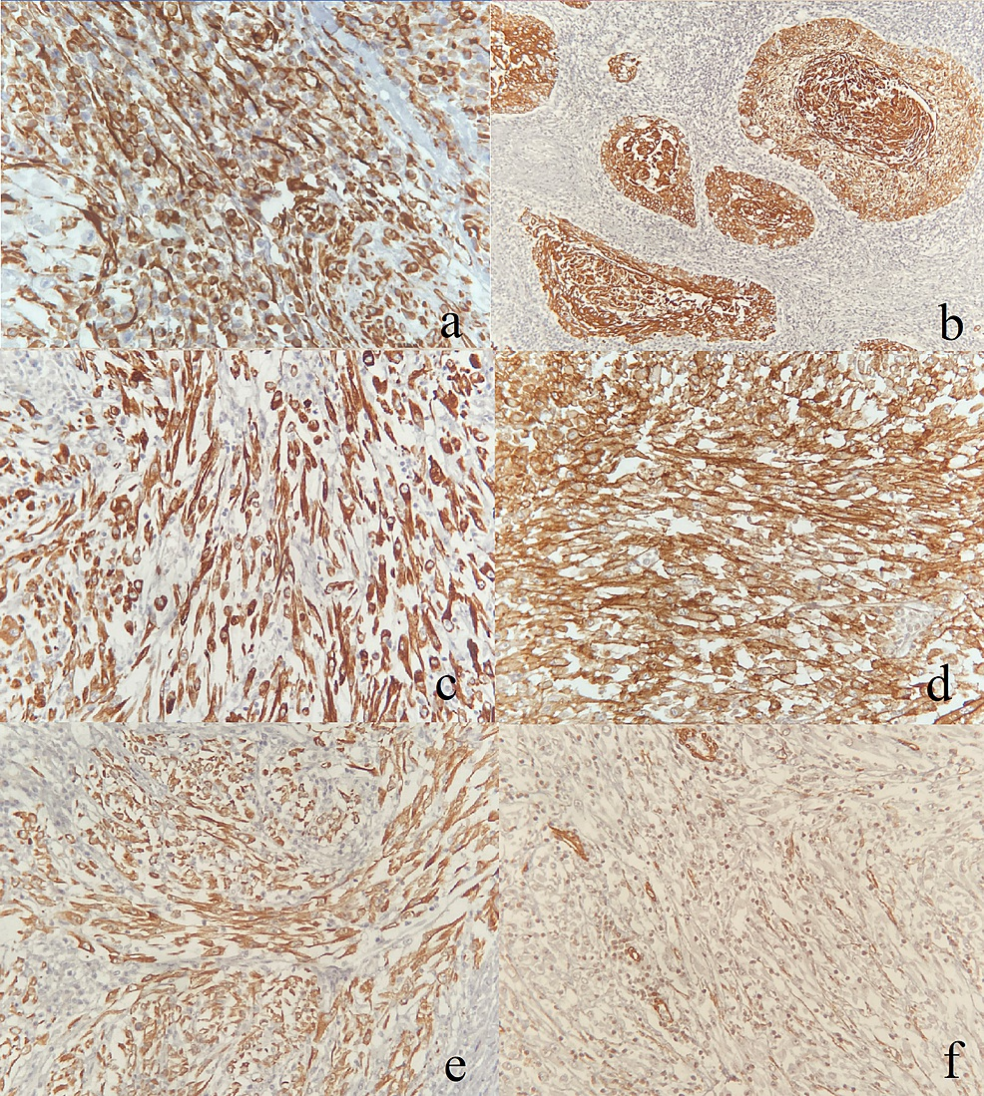
Immunohistochemistry profile. Positive for vimentin (a). CK5\6 in squamous differentiation (b). Positive for smooth muscle antigen (SMA) (c). Positive for CK7 (d). Positive for epithelial membrane antigen (EMA) (e). Negative for desmin (f).

Macroscopic examination showed a fairly-circumscribed, bulging, gray-white mass located mainly in the renal pelvis and mostly replacing the entire kidney. It measured 14 cm in maximum dimension. The mass cut sections were homogenous with areas of necrosis, with a stent present (Figure 6). The mass is away from margins and Gerota’s fascia by 2 cm. The remaining kidney parenchyma showed dilated cystic spaces containing multiple brownstones. Furthermore, the resected left renal hilum lymph node measured 7 cm in maximum dimension. Microscopically, the tumor showed urothelial carcinoma of the renal pelvis with dysplasia in the background. In addition, the tumor had a predominance of sarcomatoid differentiation. Furthermore, foci of squamous formation containing keratin formation, and other foci of glands with mucinous and goblet cells lining were seen. The tumor was focally extending to the perinephric fat. Margins were negative; however, the ureteric margin showed dysplasia. The left renal hilum lymph node was positive for metastatic carcinoma. The final diagnosis based on the examination was UC of the renal pelvis with sarcomatoid differentiation (70%) and unusual histological differentiation including squamous (25%), and glandular (<5%) (Figure 6). In addition, there was no outside pathology confirmation done. Immunohistochemistry profile was positive for CK7, epithelial membrane antigen (EMA), vimentin, and focally for smooth muscle antigen (SMA). However, it was negative for desmin, cytokeratin (AE1/3), and GATA-3 (Figure 7).

According to the American Joint Committee on Cancer (AJCC), the pathological stage is pT3N2Mx. After a couple of days, the patient underwent a cystourethrogram, and there was no leak. The patient was eventually discharged in good health the next day and was given an appointment. The plan of the oncology team was to start adjuvant chemotherapy and perform positron emission tomography-CT (PET-CT) for the suspicious distant lymphadenopathy. In addition, CT, cystoscopy, and urine cytology were supposed to be done regularly. However, the patient missed the appointment, so the current status of the patient is unknown.

## Discussion

Horseshoe kidney is considered one of the most common congenital anomalies in the kidneys. It involves the two kidneys being fused at their lower poles by the isthmus, a region consisting of fibrous tissue and parenchyma [6]. According to the current literature, renal cell carcinoma (RCC) is considered the most common type of tumor in the horseshoe kidney, with an incidence no higher than the normal population [5]. However, renal pelvis UC is more frequent in cases with horseshoe kidneys than in the normal population possibly due to the increased risk of chronic obstruction, increased prevalence of renal calculi, and recurrent urinary infection [6]. In the first large case series of tumor pathology in horseshoe kidneys of 111 cases reported by Buntley, 50% were renal adenocarcinomas; 25% were transitional cell carcinomas (TCCs), which are now called UCs; and the remaining 25% were nephroblastomas. In a more recent case series in the Japanese literature, Hayashi et al. reported 35 cases: 54.3% were adenocarcinomas, 17.1% were renal pelvis tumors, and 14.3% were nephroblastomas [7]. Currently, there are 200 reported cases of tumors in the horseshoe kidney. Balawender K et al. reported that the most common tumor detected is RCC, which constitutes 45% of the tumors, followed by Wilms and TCC, accounting for 20% of tumors in the horseshoe kidney [5].

Upper urinary tract UCs are uncommon and account for only 5%-10% of all UCs, while bladder UCs are more common and account for 90%-95%. Having a variant histology worsens the prognosis of Upper urinary tract UCs compared to pure UCs. Additionally, variants are present in approximately 25% of upper tract UCs [1]. According to a retrospective study on UC of the upper tract of 115 cases, the variants reported were squamous (7%), sarcomatoid (6%), and glandular (4%). In addition, there is only one case reported with these three histological variants in normal kidneys, emphasizing the extreme rarity of this case [8]. In our case, the patient had the same histological variants. However, to the best of our knowledge, this is the first case reported with these three histological variants in a horseshoe kidney.

Hematuria is considered the most common presenting symptom, and it is reported in 70%-80% of patients. Other less common symptoms include flank pain (20%) and flank mass (10%) [9]. In our case, the patient presented with gross hematuria and flank pain, which was highly suggestive of pyelonephritis. Moreover, urine culture was done and revealed Gram-positive bacteria. In our case, the patient went through comprehensive workups from cytology to radiological investigation and surgical pathology. Cytology revealed urothelial cells, and based on these findings, the patient underwent an MRI, which showed markedly increased hydronephrosis. Other findings included the soft tissue mass that measures 8.3 × 6.3 × 7.0 cm. Satellite lesions were also found, raising the suspicion of UC. Then, nephroureterectomy was performed, which is the gold standard treatment for all high-risk tumors. However, the existing literature on surgical approaches for nephroureterectomy of a horseshoe kidney is limited. One significant intraoperative concern of horseshoe kidneys is the identification and control of the arterial supply. It has a notoriously anomalous arterial supply, particularly to the lower poles and isthmus [9].

The sarcomatoid variant of UC of the renal pelvis is a rare type of cancer. It presents in males more than in females with a ratio of 2-3:1. The most common presentation of sarcomatoid UC is hematuria. The gross pathology reveals tumor that ranges from 2.5 to 12 cm in greatest dimension; exophytic; with a polypoid or pedunculated growth pattern; and, less often, sessile and diffusely infiltrative, with a typical appearance of areas of extensive necrosis. Microscopically, the tumor is composed of a mixture of malignant epithelial and spindle cells. The carcinoma component is most often urothelial cell carcinoma and less frequently squamous cell carcinoma, adenocarcinoma, small-cell carcinoma, or mixtures of these histological subtypes [10]. The histological distinction of sarcomatoid carcinoma and “true” carcinosarcoma is often tricky, and immunohistochemistry is a helpful diagnostic adjunct in the proper classification of sarcomatoid neoplasms. The biological characteristic of sarcomatoid UC is invasive, with a low survival rate even with active and extensive treatment. In terms of management, the vast majority of patients underwent surgery, but few studies suggested that adjuvant chemotherapy could improve the patient’s prognosis. Furthermore, no cases reported molecular targeted drug therapy and immunotherapy as a mainstay treatment. Compared with patients with pure UC, patients with a sarcomatoid variant are diagnosed at a later stage and have a worse prognosis [11]. It is difficult to compare the presentation and prognosis of sarcomatoid UC of the pelvis in a horseshoe kidney with the cases that do not have the anomaly since there are no cases of horseshoe kidneys reported with this variant. However, a further worse prognosis is anticipated due to this anomaly’s disadvantageous presentation and anatomy [6].

Squamous cell carcinoma of the upper tract is considered rare and develops most likely because of chronic inflammation and chronic infection [12]. Moreover, in our case, the patient suffered from recurrent upper urinary tract infections and stones on the same side of the tumor, which most likely can be explained by the horseshoe kidney the patient had. Because of the slow-growing nature of the tumor, it is often discovered in a late stage. It is often diagnosed using fine-needle aspiration cytology of the renal collecting system [13]. Furthermore, PET-CT can detect metastasis and prolong survival, which was suggested by the treating physician to be done for the patient [14]. Most of the papers published on this topic are case reports; thus, there is a lack of standardized treatments for such patients. Thus, most of the patients undergo radical nephroureterectomy with bladder cuff resection, as in our case. Some researchers have suggested that patients may benefit from platinum-based chemotherapy [15]. Unfortunately, it has been reported that if the tumor has squamous differentiation, it is more likely to be invasive with poor prognosis, with a five-year survival of less than 50% and 59 months as median survival time [16].

In the retrospective study of 115 cases, glandular differentiation of the renal pelvis constitutes five cases, and its incidence is unknown [8]. The glandular UC is composed of true glandular spaces, and they present under the microscope as a tubular or enteric gland with mucin production. The presence of mucin alone is not enough to diagnose glandular UC because it is also present in 60% of the urothelium of the neoplasm [17]. In our case, microscopic examination showed that the glandular differentiation constitutes less than 5% of the entire resected tumor.

The postoperative pathology staging suggested T3N2Mx using the AJCC 8th edition manual [18]. The patient presented late with this advanced stage due to the tumor’s pathological characteristics and its slow-growing nature and was only discovered incidentally after imaging for abdominal pain. The patient underwent a nephroureterectomy, which is performed on most of the patients with similar cases. Nevertheless, few studies have proposed that adjuvant chemotherapy could improve the patient’s prognosis. Hence, adjuvant chemotherapy was suggested by the treating physician to exclude distant metastasis.

## Conclusions

This pathological case of upper tract UC in a horseshoe kidney is extremely rare. Additionally, preoperative diagnosis and staging are difficult. Therefore, we wish that this case report will shed some light on the importance of early diagnosis to achieve better outcomes and curative effects for similar cases, especially in those with predisposing factors, such as a horseshoe kidney, in the upcoming years.
